# Improving Compliance and Quality of Documentation of Cerebral Function Monitoring in a Neonatal Neurocritical Care Unit

**DOI:** 10.1097/pq9.0000000000000461

**Published:** 2021-08-26

**Authors:** Ipsita Goswami, Panadda Chansarn, Jose Aldana Aguirre, Floura Taher, Diane Wilson, Cecil Hahn, Amr ElShahed, Kyong-Soon Lee

**Affiliations:** From the * Division of Neonatology, The Hospital for Sick Children, Toronto, Ontario, Canada; † Department of Neonatology, Faculty of Health Sciences, McMaster University, Hamilton, Ontario, Canada; ‡ Division of Neurology, The Hospital for Sick Children, Toronto, Ontario, Canada; § Department of Paediatrics, University of Toronto, Ontario, Canada

## Abstract

**Introduction:**

Neonates admitted to neurocritical care units frequently undergo continuous bedside cerebral function monitoring (CFM). Documentation of CFM findings that are complete and accurate can augment the quality of care through improved communication. We aimed to increase the compliance with and quality of CFM documentation in the electronic medical records by 50% in our neonatal intensive care unit over 6 months.

**Methods:**

We used the Plan-Do-Study-Act methodology, process mapping, and fishbone analysis. We implemented interventions, including the development of standardized EMR templates, face-to-face reminders at staff meetings and clinical handover sessions, and teaching on CFM interpretation.

**Results:**

We evaluated 50 and 161 charts pre (August–October 2018) and postintervention (December 2018–July 2019), respectively. We improved compliance with documentation from 72% to 89% (*P* = 0.004); and the quality of documentation from 10% to 61% (*P* < 0.001). Multimodal reminders to document and educational sessions to increase familiarity with CFM interpretation effectively improved the quality of documentation.

**Conclusions:**

We improved the compliance with and the quality of CFM documentation using targeted quality improvement interventions with case-focused educational sessions, reference tools, and standardized templates. Barriers to compliance with documentation were adverse effects on the workflow that changes in the EMR system may address. A significant challenge to sustainability was the high frequency of rotating trainees. We addressed this challenge by developing mandatory electronic teaching modules that include reminders to document and a case-focused teaching curriculum; to increase awareness of the importance of CFM documentation and increase confidence in CFM interpretation.

## Abstract 2

Supplemental Digital Content is available in the text.

## BACKGROUND

The introduction of neurocritical care to neonatal intensive care units (NICUs) has improved the care of neonates with congenital or acquired neurological illnesses.^1^ The most commonly encountered conditions in NICUs include neonatal encephalopathy secondary to perinatal injury, metabolic derangements, infections, brain malformations, vascular accidents, and prematurity.^2^ Electroencephalography (EEG) is an essential evaluation in these patients as it reflects cerebral electrical activity and is a sensitive tool for seizure detection.^3^ An adjunct to conventional EEG, amplitude-integrated EEG or cerebral function monitor (CFM), can have wider availability and be interpreted by the neonatal team with training. Moreover, CFMs have been adopted by many NICUs who may have limited access to continuous EEG monitoring.^4,5^ While CFMs are increasingly used in NICUs, standards for reporting are not well established,^6^ and interpretation of CFMs have shown poor reproducibility.^7^

With the increasing use of CFMs in our NICU, there were concerns regarding how CFMs were used for clinical decision-making. Specifically, frontline and on-call staff with varying CFM interpretation skills conducted CFM interpretations. From a quality of care and safety perspective, written medical documentation is the primary piece of evidence in determining whether clinicians provided appropriate care in a given clinical circumstance.^8^ Since incomplete documentation can result in miscommunication, inappropriate management decisions, and compromise patient safety, we undertook a quality improvement (QI) project to improve the compliance with and quality of CFM documentation.

### Specific Aims

The aim was to improve compliance with CFM documentation in the electronic medical records (EMRs) by bedside care providers in a neonatal neurocritical care unit, from a baseline of 72% to 100% within 12 months.

A secondary aim was to improve the quality of documentation from 10% to 50% within 12 months. Good quality documentation was based on the inclusion of all 3 of the elements of description of the background, description of sleep-wake cycling, and presence/absence of seizures in the preceding 12 hours.

## METHODS

### Setting

We conducted the study in the NICU at the Hospital for Sick Children in Toronto, Ontario, Canada, which serves as the quaternary NICU for a catchment area of 75,000 annual births. The NICU has 800 admissions per year, among which approximately 20 neonates per month have a primary admission diagnosis of a neurological condition. A multidisciplinary neonatal team comprised of neonatal fellows, nurse practitioners (NPs), pediatric residents, nurses, respiratory therapists, dieticians, pharmacists, and neonatologists managed the patients. The neurology service that includes neonatal neurology fellows and pediatric neurologists interested in neonatal neurology regularly consults with the NICU's neurocritical care patients.

The CFMs are stored in the Respiratory Therapy Department, which maintains a running log of all patients on CFM. A core neurocritical care team consisting of a neonatal neurology fellow and neonatal neurology NP reviews all neurocritical care patients daily during the weekdays. They review weekend events on Mondays. To detect seizures, the neurocritical care team reviewed CFMs, nursing notes, and medical orders for each patient.

## PROBLEM DESCRIPTION

A baseline audit of 50 consecutive EMRs of patients on CFM monitoring between August 1, 2018, and October 31, 2018, showed that 72% (36/50) patient charts had some documentation of CFM findings in physician or NP notes within 24 hours of initiation of CFM monitoring. The clinical designation of the person entering the note in the patient charts was fellow in 29 (58%), resident in 10 (20%), and NP in 11 (22%). All notes were in free-text format within the daily progress notes. The CFM details lacked consistency, and the terms used to describe the backgrounds were highly variable, for example, “suppressed,” “low voltage,” “normal background,” “unchanged,” “within normal limits,” “abnormal background,” and “not normal.” Most progress notes did not document artifacts or impedance checks.

## DESIGN

**Fig. 1.**
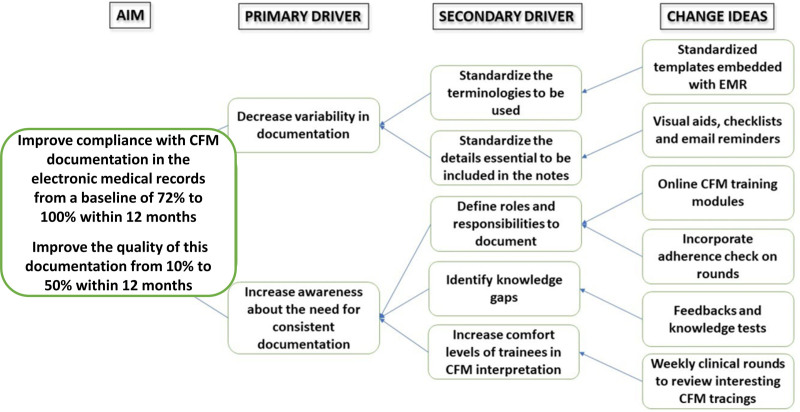
Key driver diagram showing the overall aim of the project, the primary drivers that contribute directly to achieving the aim, the secondary drivers that are components of the primary drivers, and specific change ideas to test for each secondary driver.

The QI project team consisted of 4 senior neonatal fellows, 1 NP with specialized training in neonatal neurocritical care, 1 pediatric neurologist, and 2 staff neonatologists. We prepared an Ishikawa diagram to identify the barriers to timely and accurate documentation of CFM findings (**see Appendix 1, supplemental Digital Content 1,** http://links.lww.com/PQ9/A300). Subsequently, we invited frontline staff that included neonatal nurses, respiratory therapists, and physicians. We created a swim lane diagram to provide clarity and accountability of the different NICU team members for CFM documentation (**see Appendix 2, supplemental Digital Content 1,** http://links.lww.com/PQ9/A300). We selected interventions aimed at the most common factors preventing documentation, namely lack of standardization of the CFM note, lack of awareness of the importance of CFM documentation, and lack of familiarity with CFM interpretation. Figure 1 shows our key driver diagram. The QI team met every 4 weeks to monitor the project and make changes during implementation. The target population for the interventions was residents, neonatal fellows, and NPs who had individual patient assignments and were primarily responsible for completing admission and daily progress notes. We educated clinical support nurses, bedside nurses, respiratory therapists, and neonatologists, who provided support to the frontline staff responsible for the CFM documentation about the initiative.

## INTERVENTIONS

We completed 4 Plan-Do-Study-Act cycles with the goals to introduce the new tools and templates, clarify role definitions, increase familiarity with standardized terminology for CFM documentation, and increase compliance with CFM documentation (**see Appendix 3, Supplemental Digital Content 1,** http://links.lww.com/PQ9/A300).

## PLAN-DO-STUDY-ACT CYCLE 1 (DECEMBER 2018–JANUARY 2019): DEVELOPMENT AND IMPLEMENTATION OF STANDARDIZED TEMPLATES ON EMR AND LAMINATED TOOLS

We developed stand-alone note templates to standardize CFM documentation that included the most commonly accepted terminologies for CFM documentation in the literature.^9^ These templates (**see Appendix 4, Supplemental Digital Content 1,** http://links.lww.com/PQ9/A300) were incorporated into our EMR by our information technology team using SmartPhrases. Two standardized templates were created, one for documenting CFM background as a daily note and a second for documenting any seizures detected on CFM as an event note. Additionally, we prepared CFM tool cards to act as a visual reminder of the standard terminologies for each of the CFM's typical patterns. We sent detailed instructions on how to use these templates within the EMR by email to the stakeholders. We placed laminated copies of the templates in high visibility areas within the NICU, including the common workroom where frontline staff used the computers for daily notes. We attached CFM tool cards to the CFM machines for quick reference.

## PDSA CYCLE 2 (FEBRUARY–MARCH 2019): CLARIFICATION OF ROLE DEFINITIONS FOR CFM DOCUMENTATION

There was a lack of clarity regarding who was responsible for CFM documentation; different care providers wrote multiple CFM notes for the same period. We communicated the explicit responsibilities of each of the nurses, respiratory therapists, and physicians/NPs by email to frontline staff. Project team members attended monthly meetings of allied health professionals to remind and encourage the members in their respective roles for the common goal of improving CFM documentation.

## PDSA CYCLE 3 (APRIL–MAY 2019): INCREASE FAMILIARITY WITH CFM INTERPRETATION AND MULTIMODAL APPROACH TO PROVIDE REMINDERS TO PERFORM CFM DOCUMENTATION

The majority of residents and neonatal fellows were new to the neurointensive care unit and had limited experience with CFM interpretation, and some were not comfortable interpreting the CFM. Before any interventions, we circulated a 2-part questionnaire to the stakeholders. The first part had qualitative questions for the needs assessment, and the second part had a knowledge test. To build and strengthen competency, we started weekly 10-minute teaching sessions after morning handover. We based the teaching on a CFM strip from an actual NICU patient, which emphasized the key features for clinicians to note and describe. At the end of the session, we reminded the team about the available template and showed them the steps for proper documentation on the EMR. To further emphasize the need for proper documentation, we incorporated CFM discussions into daily ward rounds. Additionally, QI team members had face-to-face interactions with the physicians/NPs responsible for completing the CFM note during their workday. They provided real-time reminders to complete the note, did bedside teaching on their patients, and sought feedback about the CFM templates and tools.

## PDSA CYCLE 4 (JUNE–JULY 2019): PROMOTE ONGOING CFM DOCUMENTATION, SUSTAINABILITY

To promote ongoing documentation, we implemented several interventions to heighten awareness regarding the importance of CFM documentation. We created online tools on “iLearn” (iLearn, Inc. Marietta, Ga.) for staff and trainees, including the new guidelines for documentation and CFM teaching modules. As bedside nurses had the most direct contact with the frontline physicians and NPs; and had a crucial role in supporting the frontline staff responsible for the CFM documentation, we administered a brief mandatory educational module for nurses. This educational module included the evidence supporting CFM use and teaching on the interpretation of common patterns and artifacts. We created a teaching file containing anonymized CFM strips and administered it to new residents and fellows during the monthly orientation session on the first day of each new rotation. We also utilized these teaching strips during weekly clinical neurology rounds attended by the NICU team, neurologists, and neuroradiologists. We used them to review CFM interpretation and teach the CFM findings during ongoing clinical care.

## MEASURES

The primary outcome was compliance with CFM documentation as determined by the proportion of charts with completed CFM documentation. The secondary outcome was improved quality of CFM documentation, as determined by the proportion of charts with complete CFM documentation based on the inclusion of all 3 of the elements of description of the background, description of sleep-wake cycling, and the presence/absence of seizure in preceding 12 hours (**see Appendix 5, Supplemental Digital Content 1,** http://links.lww.com/PQ9/A300). Our evaluation focused on documentation that occurred within the first 24 hours of initiation of CFM monitoring. This time was the most important period of use and allowed data collection to be feasible.

Process measures included the proportion of charts that staff complied with the intervention, that is, used the standardized templates for CFM documentation and completion of the seizure event note when a seizure was suspected on CFM. A balancing measure assessed user satisfaction with the project as measured by feedback from frontline personnel.

## ANALYSIS

We tracked outcome and process measures on statistical process control (SPC) charts. We collected data for the baseline period from August 2018 to October 2018 and the postintervention period from December 2018 to July 2019. We identified signals indicating special cause using standard control chart rules.^10^ We used a Chi-square test to compare proportions between baseline and postintervention periods and a *P* value <0.05 for statistical significance.

## ETHICAL CONSIDERATIONS

The Hospital for Sick Children Quality Review Committee reviewed this project and provided approval and a waiver of the Research Ethics Board review. We removed patient identifiers from all data collected and teaching cases.

## RESULTS

**Fig. 2.**
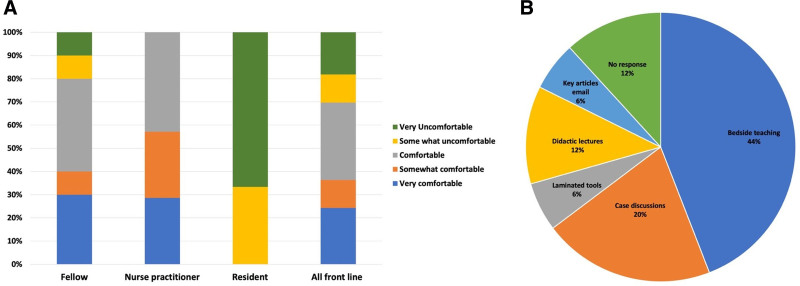
Results of needs assessment for educational intervention regarding the level of comfort with the interpretation of cerebral function monitor and preferred method of teaching. A, Stacked bar graph of comfort levels of frontline staff in bedside interpretation of cerebral function monitoring. B, Pie chart showing the methods of teaching preferred by the frontline staff for effective knowledge translation.

Among 34 frontline providers who responded to the needs assessment, there were 20 (58%) fellows, 5 (15%) residents, and 7 (21%) NPs. The level of confidence on CFM interpretation was variable by background, as shown in Figure 2A, with residents having the least confidence. The preferred teaching method by all respondents was bedside teaching and case discussions, as shown in Figure 2B.

**Fig. 3.**
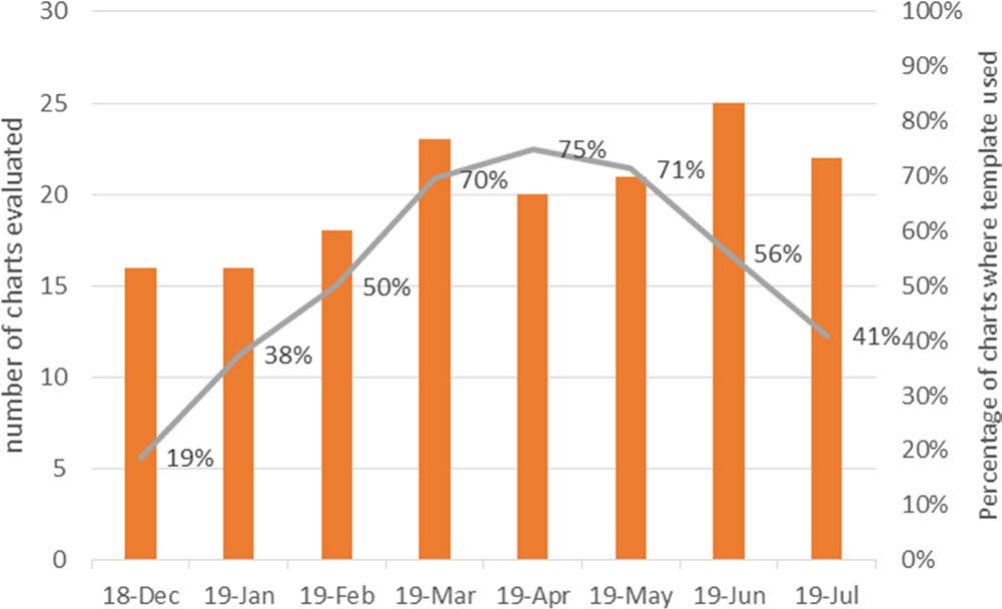
Bar and line graph showing the process measure: compliance with the use of the electronic template. The left *y* axis and the bars represent the total number of charts evaluated for each period. The right *y* axis and the line graph represent the percentage of charts where clinicians used the electronic template.

**Fig. 4.**
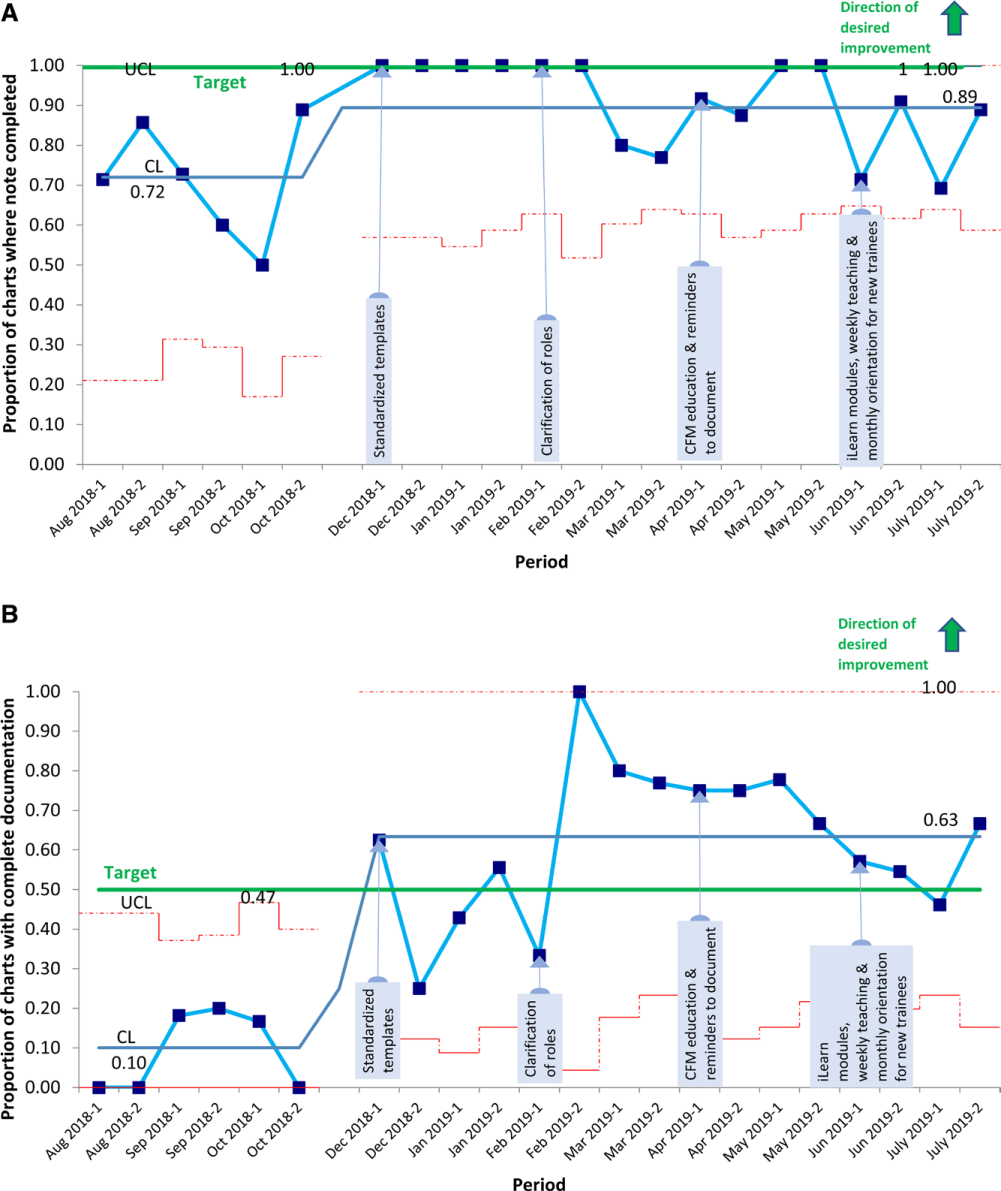
SPC p chart for outcome measures of compliance with documentation and quality of documentation. The CL represents the overall proportion during the pre and postintervention periods. Dotted red lines represent control limits. CL, centerline; LCL, lower control limit; UCL, upper control limit.Fig. 4. A, SPC p chart for outcome measure: compliance with documentation as measured by the proportion of charts where clinicians completed the note. We did not see special cause variation postintervention. B, SPC p chart for the outcome measure: quality of documentation as measured by the proportion of charts with complete documentation. We saw special cause variation postintervention.

In total, we evaluated 211 cases, 50 pre and 161 postintervention. Figure 3 shows the process measure of compliance with using the template during the project’s implementation, ranging from 19% at the beginning of the project, peaking at 75%, then decreasing towards the end of the project. Figure 4A and B shows the SPC p charts for the outcome measures of compliance with documentation and quality of documentation, respectively. For compliance with documentation, the baseline rate was 72%; we implemented interventions in November 2018, and the postintervention rate was 89%. In the SPC chart, we did not see special cause variation between the pre and postintervention periods, but the difference in overall pre and post rates was statistically significant (*P* = 0.004). For the quality of documentation, the baseline rate was 10%, and the postintervention rate was 61%; and we did see a special cause variation. In terms of the individual components of the CFM documentation, there was an improvement in all components, with the most significant improvement in the documentation of impedance from 6% at baseline to 55% postintervention (Table 1). In terms of where the documentation occurred postintervention, frontline clinicians used the CFM template in 60% of documented CFM cases. Among cases with seizures during the first 72 hours of admission, clinicians documented real-time only 25% of seizures at baseline, and this increased to 90% postintervention.

**Table 1.** Comparison of Cerebral Function Monitor Documentation in Preintervention and Postintervention Periods

	Preintervention	Postintervention	*P*
	N = 50	N = 161	
	n (%)	n (%)	
CFM documentation within 24 h	36 (72%)	143 (89%)	0.004
Template used	0 (0%)	87 (54%)	<0.001
Complete documentation	5 (10%)	99 (61%)	<0.001
Background described	13 (26%)	119 (74%)	<0.001
Standard terminology for background used	11 (22%)	117 (73%)	<0.001
Sleep wave cycling mentioned	6 (12%)	104 (65%)	<0.001
Presence/absence of seizure mentioned	24 (48%)	134 (83%)	<0.001
Impedance mentioned	3 (6%)	88 (55%)	<0.001
Any seizures during first 72 h of life	12 (24%)	51 (32%)	0.30
Seizure event note entered	3/12 (25%)	46/51 (90%)	<0.001

CFM, cerebral function monitor.

In the postproject satisfaction survey, 63% (10/16) of respondents felt that the time spent on completing the template was helpful and that we should continue using the template. When asked whether the templates provided for documentation of CFM were useful, 62% (10/16) felt they were “useful” or “very useful.” However, concerns were raised by bedside physicians that CFM teaching sessions during handover delayed clinical workflow, and they felt that one-to-one reminders were not a sustainable option.

## DISCUSSION

In this multidisciplinary QI project in a neonatal neurocritical unit, we successfully improved the quality of documentation by more than 50% from a baseline of 10% to 61% during 8 months. We created and refined tools that improved the quality of documentation. These tools included a standardized reporting template, laminated reference cards, case-based teaching strips, and iLearn modules. The tools and strategies developed during our project may be useful for other NICUs to utilize if they are in the process of implementing improved documentation of bedside evaluations of patient clinical status, such as done with the CFM. Our NICU may represent other NICUs with neurocritical patients with many rotating learners of varying skill levels. Also, many NICUs use electronic medical record systems to incorporate standardized note templates such as those implemented during our study.

Although the development of the tools was valuable, a significant key to success was the entire interprofessional team’s early involvement and engagement, including bedside nurses, rather than just the frontline medical staff. This interprofessional engagement heightened awareness of the importance of complete and accurate CFM documentation and created a common team goal.

We utilized multimodal strategies to provide reminders for documentation and educate the frontline staff on CFM interpretation. Our strategies focused on embedding any new tasks within the daily workflow, including real CFM examples for teaching to immediately follow routine handover rounds and weekly neonatal neurology rounds.

We could not effect a 50% improvement in compliance, although it did increase from 72% to 89%. This lack of the desired improvement may relate to a busy clinical environment where frontline staff had limited time to do additional documentation. The workflow that required the frontline staff to create a separate note for CFM interpretation from the progress note was time-consuming and had a significant risk of being omitted. We are working with our informatics staff to automate the generation of a CFM note for all cases with a CFM order. We are utilizing electronic capture of real-life CFM cases for teaching to increase the relevance of the learning by frontline providers.

Our data demonstrate a decrease in the degree of improvement in the quality of documentation over time. This finding highlights the importance of addressing sustainability. To this aim, we developed a revised mandatory educational curriculum. This curriculum includes a self-administered iLearn module that includes content on the importance of CFM documentation as a reminder; and a series of two 1-hour online lectures with real-life case studies. These sessions are interspersed with interactive quizzes that utilize online apps that have been positively received by neonatal-perinatal fellows and neonatal staff.

## CONCLUSIONS

We were able to improve the quality of CFM documentation in a neonatal neurocritical unit using QI methodology. Critical elements for success were the multidisciplinary team’s engagement and the development and implementation of multimodal tools and interventions. Like many units with multiple rotating learners, we identified that sustainability in improvement as a significant challenge. This insight has led to ongoing QI work to refine the educational materials to focus on sustainability.

## DISCLOSURE

The authors have no financial interest to declare in relation to the content of this article.

## ACKNOWLEDGMENTS

We would like to acknowledge Carol McNair, NP, SickKids, and the bioinformatics team at SickKids for their assistance in incorporating the templates in the EMRs system.

## Supplementary Material


