# Supplementary material for: Improving Compliance and Quality of Documentation of Cerebral Function Monitoring in a Neonatal Neurocritical Care Unit
Source: Pediatr Qual Saf. 2021 Aug 26;6(5):e461. doi: 10.1097/pq9.0000000000000461 (PMC8389948; doi:10.1097/pq9.0000000000000461)
Supplement: Supplementary file 1 [file pqs-6-e461-s001.pdf]

Appendix 1. Ishikawa diagram to display the causes contributing to poor compliance with and poor quality of documentation

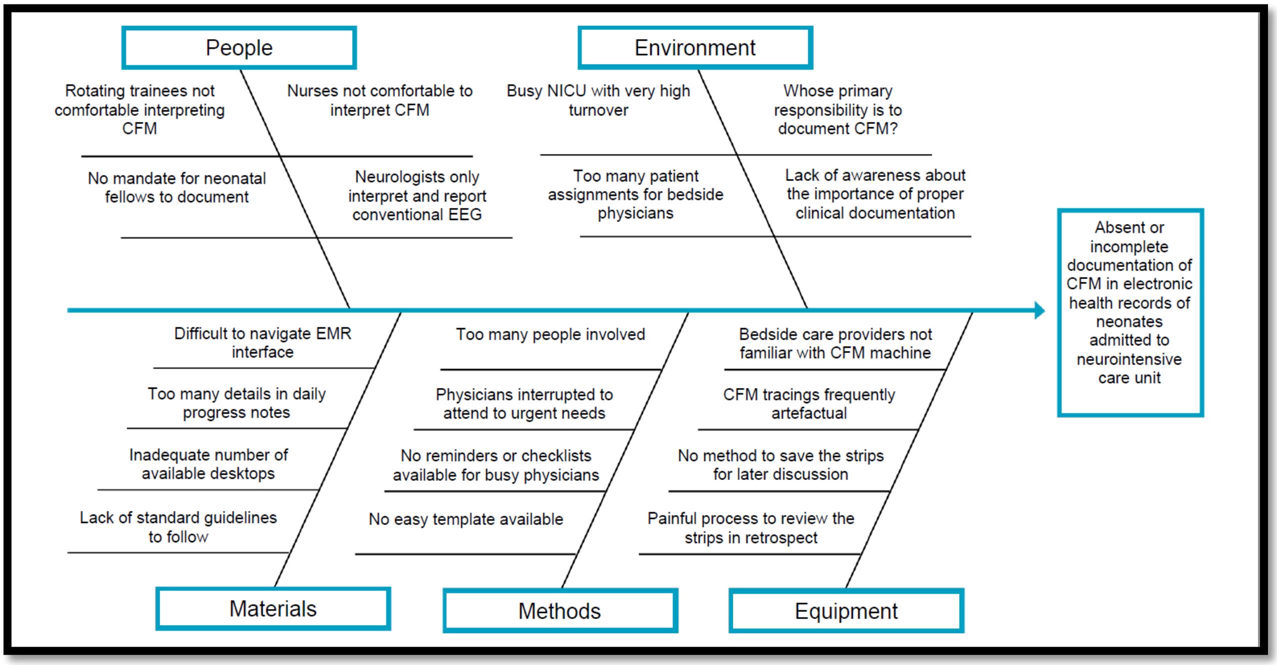

Appendix 2. Swim lane process map to demonstrate the steps toward completion of a note during the preintervention phase

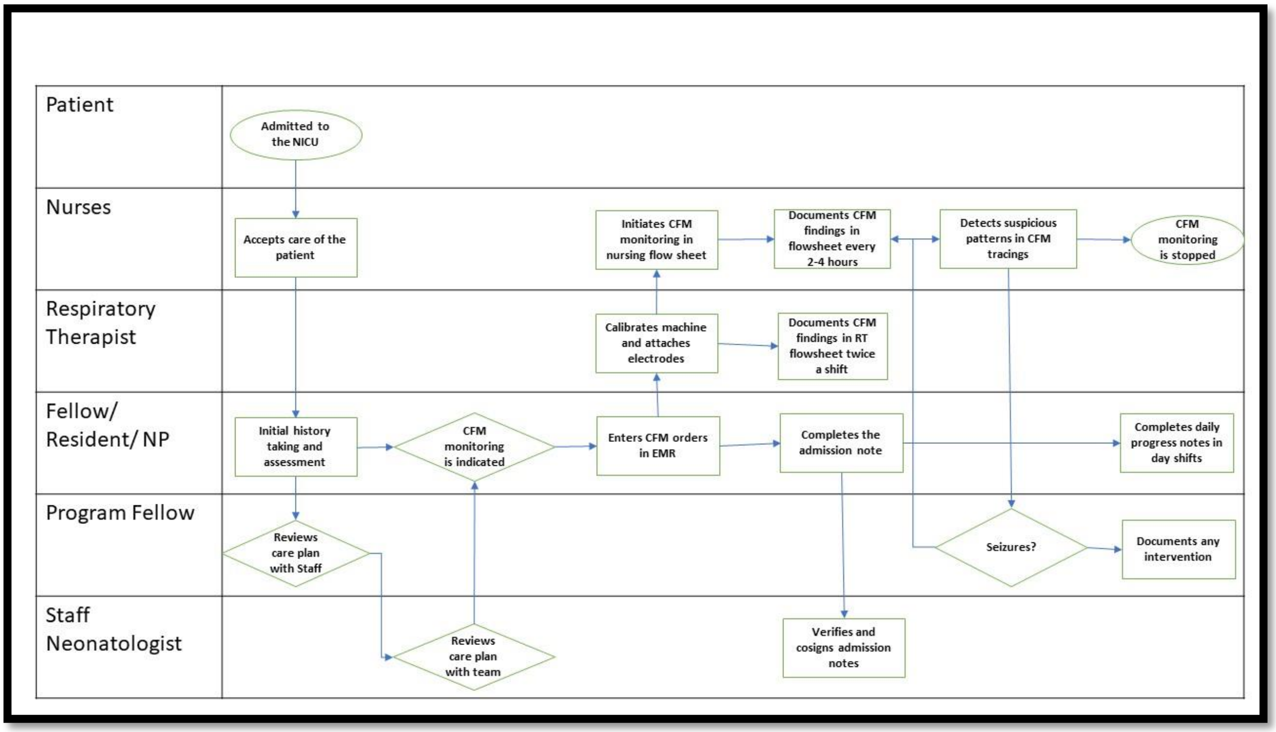

Appendix 3. Summary of Plan-Do-Study-Act cycles

Cycles	Time	Plan	Do	Study	Act
**PDSA 1**	Dec -Jan 2019	Developed and implement stand-alone, standardized note	Distributed instructions to use these templates through email	Compliance in using template was poor	Visual aids, weekly email reminders, EMR prompts
**PDSA 2**	Feb-Mar 2019	Defined the primary responsibilities of nurses, RTs and MD/NPs in documentation	Emails were circulated, focused group meetings	Informal feedback, ongoing chart audits	Weekly email reminders, development of online survey, one to one reminders
**PDSA 3**	April-May 2019	Developed teaching modules to improve comfort levels of rotating trainees	Distributed online survey for needs assessment, 10 min handover teaching session	Evaluation of online survey, ongoing chart audits	Developing more sustainable methods of education and reminders
**PDSA 4**	June-July 2019	Updating online teaching portal	Weekly neurology rounds, resident orientations	Postintervention audits of 50 patient charts	Planning for second phase

Appendix 4. Standardized templates for Neonatal aEEG note and Seizure event note on electronic medical records

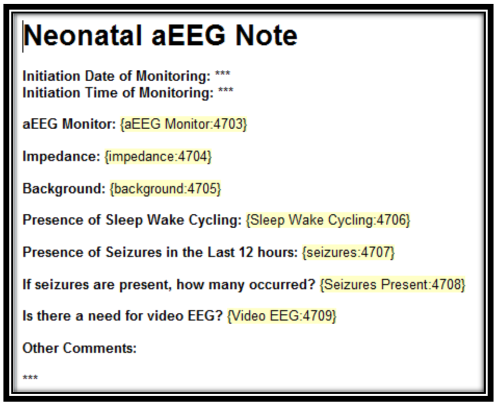
Neonatal aEEG Note

Initiation Date of Monitoring: ***
Initiation Time of Monitoring: ***

aEEG Monitor: {aEEG Monitor:4703}

Impedance: {impedance:4704}

Background: {background:4705}

Presence of Sleep Wake Cycling: {Sleep Wake Cycling:4706}

Presence of Seizures in the Last 12 hours: {seizures:4707}

If seizures are present, how many occurred? {Seizures Present:4708}

Is there a need for video EEG? {Video EEG:4709}

Other Comments:

***

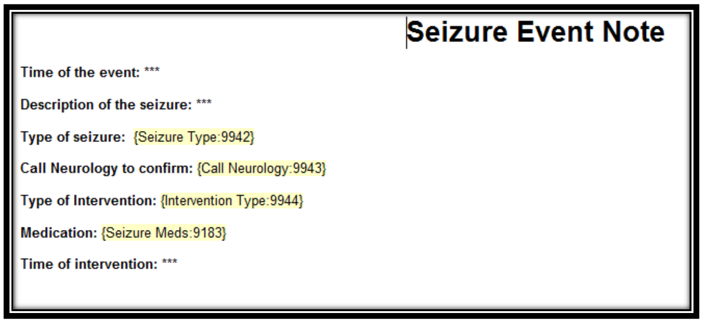
Seizure Event Note

Time of the event: ***

Description of the seizure: ***

Type of seizure: {Seizure Type:9942}

Call Neurology to confirm: {Call Neurology:9943}

Type of Intervention: {Intervention Type:9944}

Medication: {Seizure Meds:9183}

Time of intervention: ***

Appendix 5. Endpoint measures with definitions

**Outcome measures**	**Process measures**
Charts with complete CFM documentation (all 3 present):<br>a. Description of the background<br>b. Description of sleep wake cycling<br>c. Presence/absence of seizure in preceding 12 hours	Charts with CFM documentation within 24 hours
Charts where background was described	Charts where daily progress notes was used for CFM documentation
Charts where sleep wake cycling was mentioned	Charts where template was used for CFM documentation
Charts were presence/absence of seizure was mentioned	Charts where seizure event note was used in cases with a CFM detected seizure
Charts where impedance was mentioned	
Charts where standardized terminology for background was used (any one of the following):<br>a. Continuous normal voltage<br>b. Discontinuous normal voltage<br>c. Burst suppression<br>d. Low voltage<br>e. Isoelectric	

CFM: cerebral function monitor
